# Supplementary material for: Double exchange interaction in Mn-based topological kagome ferrimagnet
Source: Commun Phys. 2024 Oct 26;7(1):350. doi: 10.1038/s42005-024-01838-9 (PMC11512815; doi:10.1038/s42005-024-01838-9)
Supplement: Supplementary file 2 — Supplementary Material [file 42005_2024_1838_MOESM2_ESM.pdf]

Supplementary Material

Double Exchange Interaction in Mn-based Topological Kagome Ferrimagnet

Jiameng Wang[1, 2], Arthur Ernst[3, 4, *], Victor N. Antonov[5], Qi Jiang[6, *], Haoji. Qian[7], Deyang Wang[1, 2], Jiefeng Cao[8], Fangyuan Zhu[8], Shan Qiao[1, 2, 9, *], Mao Ye[8, 1, 2, *]

[1]State Key Laboratory of Functional Materials for Informatics, Shanghai Institute of Microsystem and Information Technology, Chinese Academy of Sciences, Shanghai 200050, People's Republic of China

[2]Center of Materials Science and Optoelectronics Engineering, University of Chinese Academy of Sciences, Beijing 100049, People's Republic of China

[3]Institute for Theoretical Physics, Johannes Keppler University Linz, Altenberger Strasse 69, A-4040 Linz, Austria

[4]Max-Planck-Institut fur Mikrostrukturphysik, Weinberg 2, D-06120 Halle, Germany

[5]G.V. Kurdyumov Institute for Metal Physics of the N.A.S. of Ukraine, 36 Academician Vernadsky Boulevard, UA-03142 Kyiv, Ukraine

[6]Center for Transformative Science, ShanghaiTech University, Shanghai 201210, People's Republic of China

[7]Research Center for Intelligent Chips and Devices, Zhejiang Lab, Hangzhou 311121, China

[8]Shanghai Synchrotron Radiation Facility, Shanghai Advanced Research Institute, Chinese Academy of Sciences, Shanghai 201204, China

[9]China School of Physical Science and Technology, ShanghaiTech University, Shanghai 201210, People's Republic of China

*Corresponding author. Email: Arthur.Ernst@jku.at
jiangqi1@shanghaitech.edu.cn
qiaoshan@mail.sim.ac.cn
yem@sari.ac.cn

Supplementary Note 1: Single Crystal Characterization

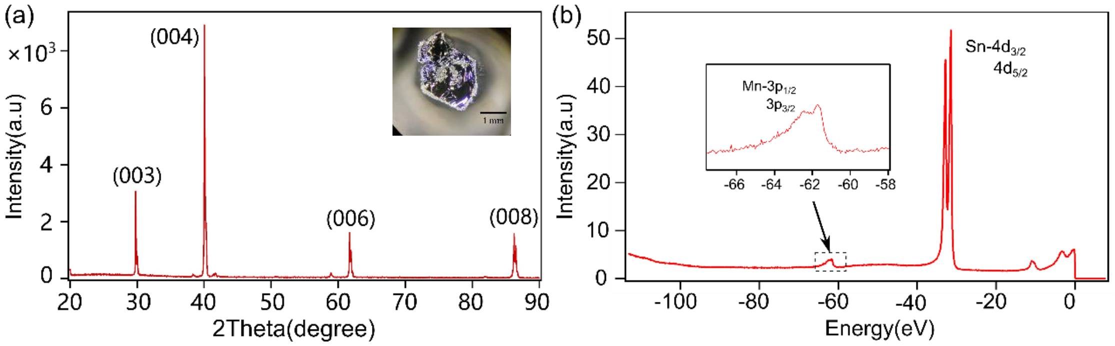

Supplementary Fig. 1 Single Crystal Characterization. (a) $GdMn_6Sn_6$ single crystal under optical microscope and XRD pattern [1]. The sharp peaks indicate the high crystallinity of the sample. **(b)** Core-level spectrum of $GdMn_6Sn_6$, with Mn-3p and Sn-4d orbitals are clearly observed.

We present the core-level photoemission intensity plot of $GdMn_6Sn_6$ obtained using Angle-Resolved Photoemission Spectroscopy (ARPES) in Shanghai Synchrotron Radiation Facility (SSRF). The clear observation of characteristic peaks of Mn-3p and Sn-4d orbitals indicate the high quality of the crystal.

Supplementary Note 2: k_z Dispersion

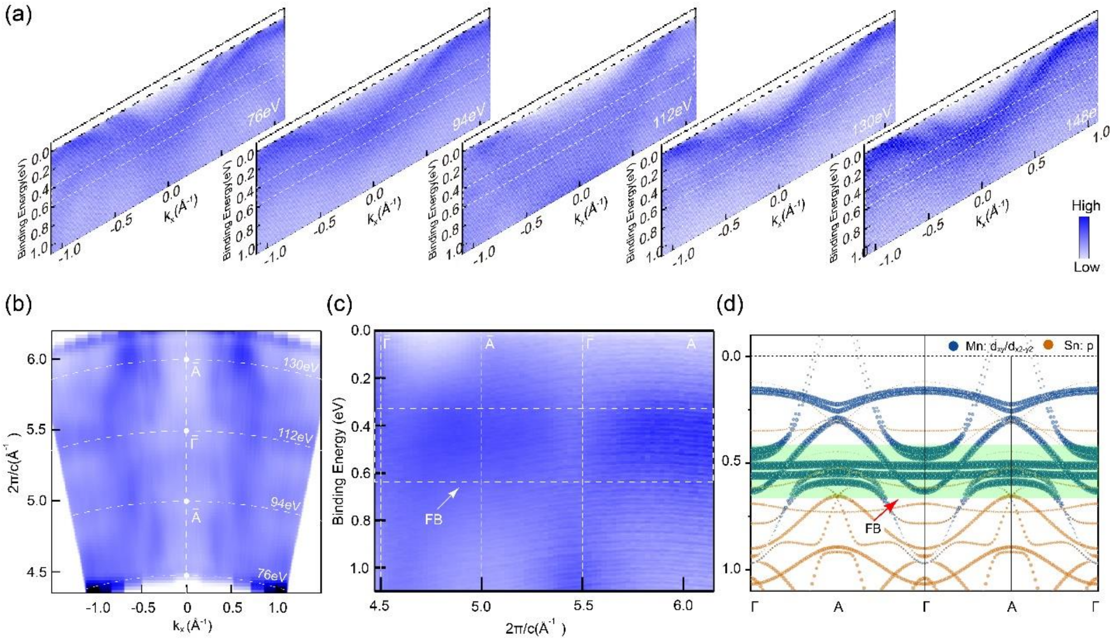

Supplementary Fig. 2 k_z dispersion measurement in $GdMn_6Sn_6$. (a) Photoemission intensity plots of $GdMn_6Sn_6$ with variable photon energies along $\bar{M}-\bar{K}-\bar{\Gamma}-\bar{K}-\bar{M}$ direction, in which the flat band locates at the binding energy of about 0.4-0.6 eV; **(b)** ARPES 2D map in $k_{/\!/}-k_z$ plane. The photon energy range used was from 70 to 150 eV, approximately covering two Brillouin zones (BZs); **(c-d)** Experimental and calculated band structure along $\Gamma-A$ direction, respectively. The blue and yellow dashed lines represent Mn d and Sn p electrons, respectively.

We conducted ARPES measurements of variable photon energy along the $\bar{\Gamma}-\bar{A}$ direction, with photon energies ranging from 70 to 150 eV, covering two BZs. From the ARPES cuts along the high-symmetry planes, it is clearly visible that the band dispersion shows almost no variation with different photon energies. The flat band along the $\bar{\Gamma}-\bar{A}$ direction is also clearly observed, appearing 0.4-0.6 eV below the E_F, consistent with theoretical calculations, which originates from the $d_{xy}/d_{x^2-y^2}$ orbitals of kagome-Mn atoms.

Supplementary Note 3: Fermi Surface

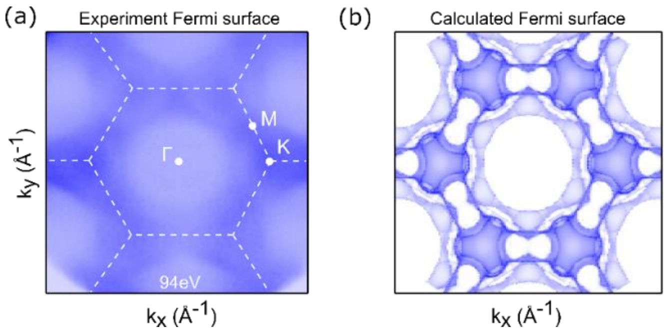

Supplementary Fig. 3. **Experimental and calculated Fermi surface of the bulk states** [2-4].

We measured the Fermi surface of $GdMn_6Sn_6$ at $h\nu = 94$ eV, and it is consistent with the Fermi surface obtained from WannierTools calculations [5]. The Fermi surface exhibits six-fold symmetry, consistent with the $GdMn_6Sn_6$ crystal structure.

Supplementary Note 4: Band Structure

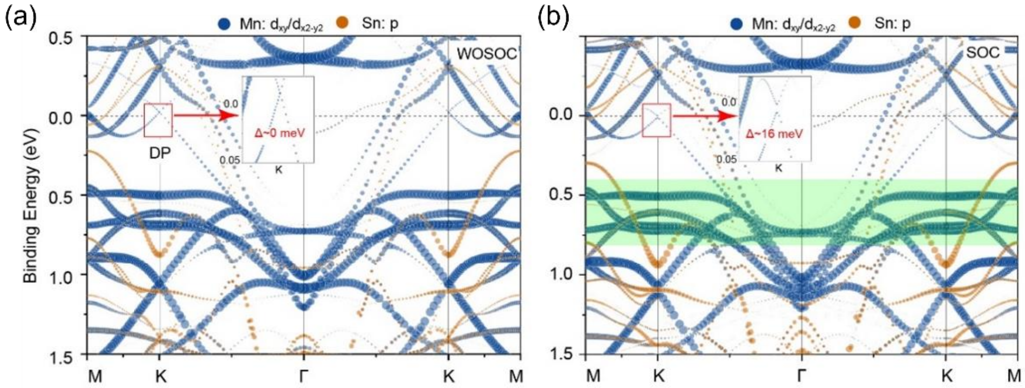

Supplementary Fig. 4 Calculated bulk band structure with Mn-d and Sn-p orbital contributions along $M-K-\Gamma-K-M$ high symmetry directions [2-4]. **(a)** Band structure without spin-orbit coupling (SOC). **(b)** Band structure with SOC, where a 16 meV gap opens at the Dirac point located at the K point. Blue circles represent Mn d orbitals, while orange circles represent Sn p orbitals.

We used VASP to calculate the bulk state energy bands with orbital resolution of SOC/WOSOC along the $M-K-\Gamma-K-M$ direction. The blue and orange circles represent Mn-d orbitals and Sn-p orbitals, respectively. The flat band is located approximately 0.5 eV below the Fermi surface and is mainly contributed by the $d_{xy}/d_{x^2-y^2}$ orbitals of Mn. Meanwhile, there is an overlap and hybridization between Sn-p electrons and the Mn-d electrons at deeper binding energy. Due to the relatively small SOC strength, it has almost no effect on the overall band structure, but it opens a 16 meV gap at the Dirac point.

Supplementary Note 5: Tight-Binding Model

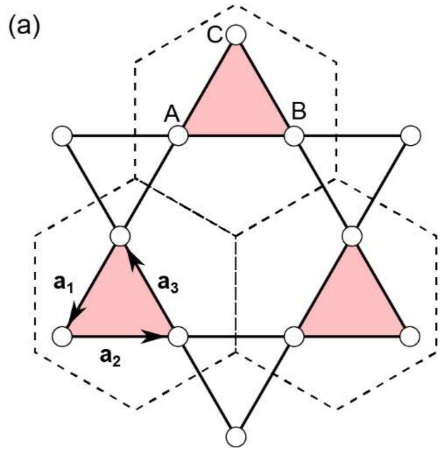

Supplementary Fig. 5 The sketch of tight-binding model in kagome lattice with NN in-plane hopping.

The structure of the kagome lattice is depicted in Fig. S4. Each unit cell (dotted hexagon) contains three inequivalent sites denoted A, B and C. We take lattice vectors as $\vec{a}_1$, $\vec{a}_2$ and $\vec{a}_3$:

$$\vec{a}_1 = \left(-\frac{1}{2}, -\frac{\sqrt{3}}{2}\right)\vec{a}$$

$$\vec{a}_2 = (1,0)\vec{a}$$

$$\vec{a}_3 = \left(-\frac{1}{2}, \frac{\sqrt{3}}{2}\right)\vec{a}$$

Considering the nearest neighbor hopping t, the tight-binding Hamiltonian is:

$$H = -t \sum_{<ij>} (e^{i\varphi_{ij}} c_i^+ c_j + H.c.)$$

Where $c_i(c_i^+)$ is the annihilation (generation) operator in the i-th lattice sites:

$$c_{i\alpha} = \frac{1}{\sqrt{N}} \sum_k e^{+ikR_i} \cdot c_{k\alpha}$$

$$c_{i\alpha}^+ = \frac{1}{\sqrt{N}} \sum_k e^{-ikR_j} \cdot c_{k\alpha}^+$$

$< ij >$ is the pair of NN lattice points, H.c. stands for complex conjugate. Hamiltonian H can be diagonalize in matrix form:

$$H = -2t \begin{pmatrix} 0 & \cos(k\vec{a}_1) & \cos(k\vec{a}_3) \\ \cos(k\vec{a}_1) & 0 & \cos(k\vec{a}_2) \\ \cos(k\vec{a}_3) & \cos(k\vec{a}_2) & 0 \end{pmatrix}$$

The solved energy eigenvalues are:

$$\varepsilon_{(1,2)} = t\left[-1 \pm \sqrt{3 + 2\cos^2(kx) + 2\cos^2(kx - \sqrt{3}ky) + 2\cos^2(kx + \sqrt{3}ky)}\right]$$

$$\varepsilon = 2t$$

The energies of this Hamiltonian (its eigenvalues) in *k*-space can be analytically determined. Furthermore, the PythTB package allows for the direct computation of the kagome lattice's band structure from the Hamiltonian in real space. This band structure is illustrated in Fig. 1(c).

Supplementary Note 6: Density of States

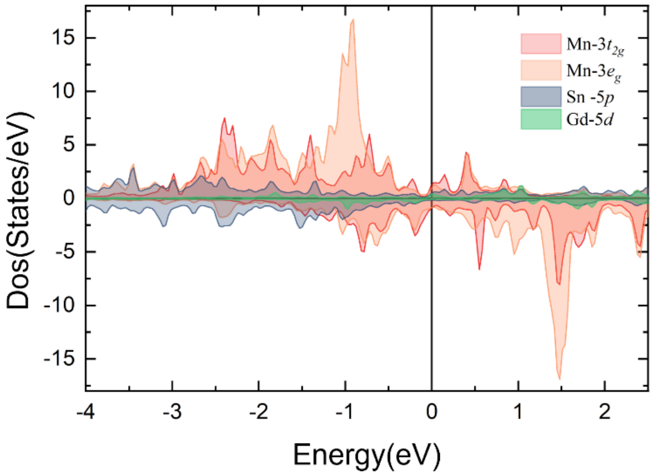

Supplementary Fig. 6 Calculated spin-polarized DOS of $GdMn_6Sn_6$.

We extracted the density of states (DOS) results from the first-principles calculations. The magnetic moment of $GdMn_6Sn_6$ mainly arises from its half-filled Mn $3d$ electrons. Notably, the peak of the majority spin electrons in Mn is observed at 1 eV below E_F. Owing to the crystal field effect, the Mn $3d$ electrons are split into e_g and t_{2g} orbitals.

Reference

[1] Asaba, Tomoya, *et al*. Phys. Rev. Lett **101**.17 (2020): 174415.

[2] Kresse, Georg, and Jürgen Furthmüller. Phys. Rev. B **54**.16 (1996): 11169.

[3] J. P. Perdew, K. Burke, and M. Ernzerhof, Phys. Rev. Lett. **77**.18 (1996): 3865

[4] V. I. Anisimov, J. Zaanen, and O. K. Andersen, Phys. Rev. B **44**, 943 (1991).

[5] Q. Wu, S. Zhang, H.-F. Song, et al., Computer Physics Communications **224**, 405 (2018).

[6] M. D. Watson, I. Markovi´c, F. Mazzola, *et al*., Phys. Rev. B **101**, 205125 (2020).
