# Supplementary material for: Double exchange interaction in Mn-based topological kagome ferrimagnet
Source: Commun Phys. 2024 Oct 26;7(1):350. doi: 10.1038/s42005-024-01838-9 (PMC11512815; doi:10.1038/s42005-024-01838-9)
Supplement: Supplementary file 3 — Description of Additional Supplementary File [file 42005_2024_1838_MOESM3_ESM.pdf]

Description Of Additional Supplementary File

File name: Supplementary Data

Description: Raw data for all figures
